# Supplementary material for: Incidence, predictors and re-treatment outcomes of recurrent myopic choroidal neo-vascularization
Source: PLoS One. 2022 Jul 21;17(7):e0271342. doi: 10.1371/journal.pone.0271342 (PMC9302801; doi:10.1371/journal.pone.0271342)
Supplement: Attachment — Submitted filename: REVIEWER COMMENTS REPLY.docx [file pone.0271342.s001.docx]

|  | **Reviewer Comments** | **Response** | **Modifications** |
| --- | --- | --- | --- |
| 1 | Was the axial length calculated at each visit, the continued increase in axial length over a period of time could be one factor for recurrence | Thank you for your comments. | Continued increase in axial length is a un-explored domain in mCNV. We found on multivariate analysis that spherical equivalence was not a predictor of recurrence. Since it was a retrospective study, we don’t have axial length measurement data as this is not part of routine follow-up. We have included this as a limitation. |
| 2 | Was FFA done before cessation of Anti-VEGF treatment? Without FFA it would be difficult to say that the membranes was inactive | Thank you for your comments. | Certainly FFA would be idea. In real world settings in the present study, membrane inactivity was decided based on OCT features and vision stabilization. |
| 3 | Quantitative profile of the mCNM could have given more information. Did the patients undergo OCTA | Thank you for your comments. | Since we had recently acquired OCTA technology, only for a handful of cases had OCTA. Moreover, getting scans of good quality was difficult due to fixation loss, background maculopathy and long axial length. |
| 4 | Did the area of mCNV of FFA have a role in the recurrence? | Thank you for your comments. | ­Unfortunately, we have FFA for less than 25% of cases at baseline. Therefore, it was difficult to analyse based on small sample and was not performed. Certainly a future prospective study with FFA and OCTA quantitative analysis needs to planned based on our results. We have included this as a limitation of the present study. |
